# Regeneration of meniscal avascular zone using autogenous meniscal fragments in a rabbit model

**DOI:** 10.1186/s12893-022-01663-3

**Published:** 2022-05-28

**Authors:** Yan Deng, Zuo-Ming Tong, Zhu Dai, Zhi-Wei Chen

**Affiliations:** 1 Department of Orthopaedics, Loudi Central Hospital, No.51 Changqing Central Street, Lou Xing District, Loudi, 417000 Hunan China; 2 Department of Orthopaedics, First Affiliated Hospital of South China University, NO.69 Chuangshan Road, Hengyang, 421000 Hunan China

**Keywords:** Autogenous meniscal fragments (AMF), Meniscal avascular zone, Meniscal regeneration

## Abstract

**Background:**

To investigate the effects of autologous meniscus fragment (AMF) implantation on injury in the meniscal avascular zone in mature rabbits.

**Methods:**

Adult New Zealand white rabbits were randomly divided into two groups. Massive one-piece meniscus tissue was implanted in situ as control. In the experimental group, AMF was used to repair the meniscal injury in the avascular zone. Meniscal damage was assessed by gross observation of the degree of healing and histological semi-quantitative evaluation within 12 weeks postoperatively. The healing of meniscus interface was assessed by gross observation semiquantitative scoring and microscopic examination hematoxylin and eosin (H&E) staining at 2, 4, 8, and 12 weeks after surgery. The expressions of proliferating cell nuclear antigen (PCNA), collagen type I (COL1A1), and collagen type II (COL2) were detected by immunohistochemical staining.

**Results:**

The degree of healing in the AMF group showed a significant increase over time (*P* < 0.05); the AMF group showed higher gross scores than the control group at 4, 8, and 12 weeks after surgery (*P* < 0.05). The histological scores in the AMF group were significantly higher than those in the control group at 4, 8, and 12 weeks after surgery (*P* < 0.05). The protein expression of PCNA in the AMF group was greater than that in the control group at 2, 4, and 8 weeks after surgery (*P* < 0.05). In addition, compared with the control group, the protein levels of COL1A1 and COL2 were significantly upregulated at each time-point. At 2 and 4 weeks after surgery, the expression level of COL1A1 increased in both groups followed by a gradual decrease after 8 weeks (*P* < 0.05). At 2, 4, 8, and 12 weeks after surgery, the expression levels of COL2 showed a gradual decrease in both groups (*P* < 0.05).

**Conclusions:**

Our study demonstrated that the AMF method can promote the repair of rabbit meniscal injury in the avascular zone, and this method may potentially be used for clinical application.

**Supplementary Information:**

The online version contains supplementary material available at 10.1186/s12893-022-01663-3.

## Background

Meniscus is a smooth, lustrous, tough and elastic fibrocartilage structure located between the tibial plateau and the articular surfaces of the femoral condyles in the knee joint [1]. The functions of meniscus include absorbing shock, reducing stress, coordinating and improving joint stability, limiting knee joint over-extension or over-flexion, promoting synovial circulation and lubrication of joints [2]. Anatomical studies have shown three distinct regions in the meniscus with different cell populations and characteristics of extra cellular matrix (ECM). In particular, the inner two thirds of the meniscus is rich in chondrocyte-like cells with poor healing capacity [2]. Thus, partial meniscectomy has been the most common strategy for treatment of meniscal injury, especially those in the meniscal avascular zone [3]. However, biomechanical and clinical studies have shown increased joint load and articular cartilage degeneration after knee meniscus resection [4]. A study by Robbins et al. demonstrated differences between OA subtypes with respect to the disease characteristics that may impact disease progression [5]. Another study reported approximately 25–50% lower risk of medical consultation for knee OA after meniscus repair as compared to arthroscopic partial meniscectomy [6]. Therefore, preserving the integrity of the meniscal structure, as much as possible, is a key imperative to avoid articular cartilage degeneration and osteoarthritis after partial meniscectomy. Allogeneic meniscus transplantation has been performed for the last more than 20 years. It is the mainstream choice for the treatment of early joint pain and osteoarthritis after total meniscus resection [7, 8]. However, the disadvantages of use of meniscus allografts include risk of disease transmission (HIV, hepatitis), graft structure changes, and host tissue integration [9]. Wider application of artificial meniscus replacement materials also requires more robust data from further clinical trials [10, 11]. The Collagen Meniscus Implant (CMI, Ivy Sports Medicine, Montvale, New Jersey, USA) is a meniscal scaffold made of collagen type I fibers (purified from bovine Achilles tendon) that has successfully replaced partial loss of medial meniscal tissue in humans. In addition, the Actifit implant (Orteq Bioengineering, London, UK) is a aliphatic polyurethane meniscal scaffold [12, 13]. Zaffagnini et al. showed that arthroscopic collagen meniscus implantation (CMI) for partial lateral meniscal defects can decrease knee pain; however, in their study the MRI scans demonstrated a decreased implant size relative to normal meniscus [14]. Various in vivo experiments to enhance the healing potential of the avascular meniscus area have been reported; these include rasping the meniscal surface and the synovial membrane around the meniscus [15, 16], adding various growth factors [17], and establishing vascular access [18]. However, these techniques are not universally applied and are still at the research stage. In addition, there is limited benefit of use of growth factors alone to promote healing of the meniscus in the avascular zone owing to their short half-life [19]. Meniscus tissue engineering is currently a hotspot treatment; however, it is still in the research stage. Moreover, the engineering technique is complex and expensive since it requires in vitro digestion, proliferation, and re-introduction of seeding cells such as mesenchymal stem cells (MSCs) and meniscal cells into the receptor after the composite stent [20]; this is a major barrier to its wider clinical application. Moreover, meniscal cells are terminally differentiated cells with extremely weak proliferative ability; these cells can only survive in vitro for a short period of time. In addition, in vitro cultured meniscal cells or MSCs tend to lose their phenotypic characteristics, secretory activity, and potential for cartilage regeneration [21–24]. Thus, development of novel strategies for the treatment of injury in the meniscal avascular zone is a key imperative.

Recently, use of autogenous meniscal fragments (AMF) for meniscal repair was shown to achieve biological and mechanical goals. Kobayashi et al. wrapped AMF into a fascial sheath to treat tears in the anterior third portion of the medial meniscus in a rabbit model; the implanted meniscal fragments helped form a meniscus-shaped tissue with appropriate stiffness in the regenerative region [25]. However, no study has investigated the application of autogenous meniscal fragments for the treatment of injury in the meniscal avascular zone. We have previously conducted a large number of studies involving in vitro organ culture for repair of injury in the avascular regions and on the biological activity of subcutaneous implantation of meniscus fragments of different sizes in rabbits. In the study by Dai et al., implantation of juvenile allograft and minced meniscal fragments was found to increase the healing of avascular meniscal injury in vitro [26]. Another study by Dai et al. demonstrated an inverse relationship between the migratory, metabolic, and proliferative abilities of rabbit fibrochondrocytes and meniscal fragment size [27]. In the present study, we aimed to investigate the effects of AMF implantation on meniscal injury in the inner 2/3 of the body and anterior horn of the lateral meniscus avascular zone in a rabbit model (Additional file 1: Fig. S1). Gross observation of the degree of healing, H&E staining, and immunohistochemistry staining for proliferating cell nuclear antigen (PCNA), collagen type I (COL1A1), and collagen type II (COL2) was evaluated for up to 12 weeks.

## Methods

### Ethical approval

The experiment was performed with the approval of the Ethics Review Committee of South China University (reference no. nhfy2010020). Care for the animals in this study was undertaken in accordance with the Guiding Directives for Humane Treatment of Laboratory Animals, issued by the National Ministry of Science and Technology on 13 September 2006.

### Animal design

All New Zealand rabbits were provided by the Animal Experiment Center of the University of South China (Hengyang, China). All rabbits were kept in the experimental animal department for 2–4 weeks to allow adaptation to the environment before performing experimental operations. Thirty-two New Zealand rabbits (age: 6 months; weight: 2.6–3.5 kg) were included in the study. All rabbits received a standard laboratory diet and water. Both knee joints were subjected to surgery in order to minimize the differences in experimental results. We selected staged surgery in the lateral meniscus of bilateral knee joint in rabbits. The time and location of the operation was marked. The normal activity of the rabbits’ post-operation was assessed by observing whether there is any gait abnormality such as claudication in the knee joint of the operation side. After the first surgical wound had healed well and the diet and health of the rabbit was normal, the contralateral side was operated at the selected time. The 64 knees were randomly divided into two groups: the control group and the autogenous meniscal fragments (AMF) group. Rabbits were sacrificed (by intravenous serazine over-dose) at 2, 4, 8, and 12 weeks after surgery for evaluation of gross morphology, histology, and biology of the regenerative meniscal tissue.

### Surgical procedure

**Fig. 1:**
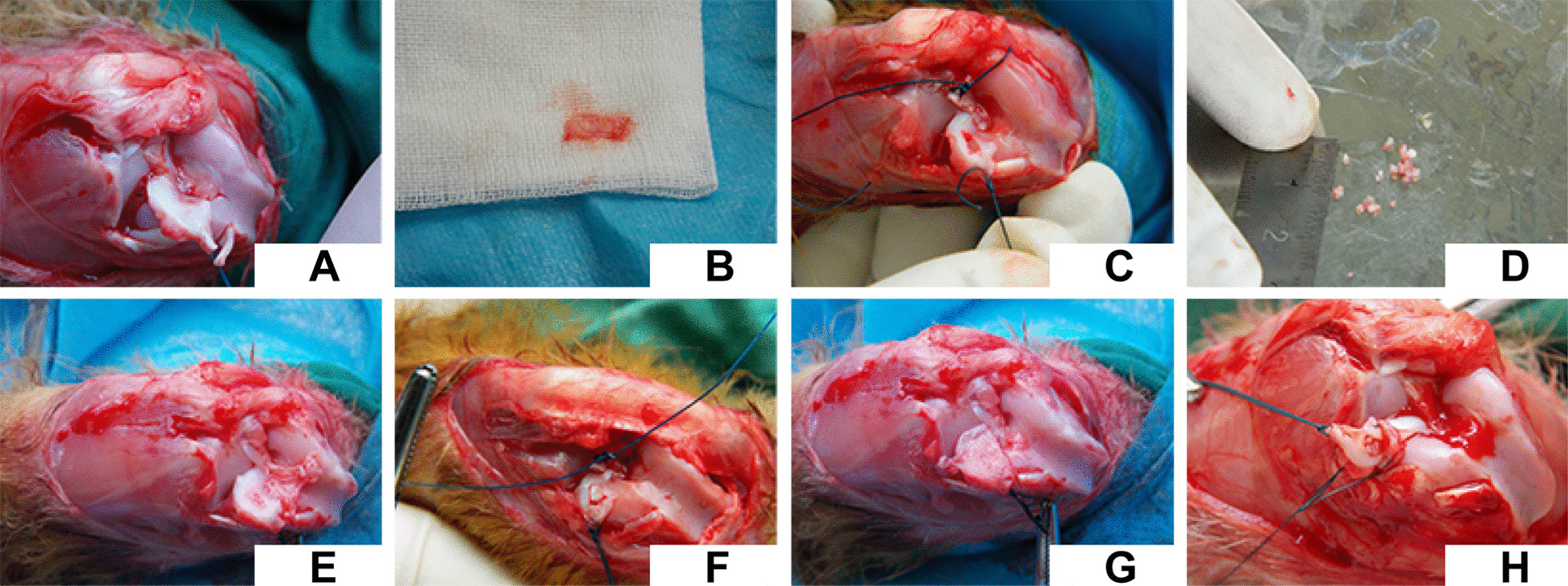
Surgical procedure for establishing a rabbit model of defect of meniscal avascular zone. **A** Exposure of the lateral meniscus; **B** removed myofascial; **C** defects of meniscal avascular zone; **D** processed meniscal tissue fragments; **E** transplantation of autologous fragments in situ into the defect (the autogenous meniscal fragments, AFM group); **F** monolithic meniscus is filling the defect (control group); **G** myofascial wrapping; **H** suturing the myofascial after wrapping

The surgical procedure is shown in Fig. 1. Intramuscular injection of penicillin (200,000 units) was administered 30 min before the operation. Anesthesia was performed by intramuscular injection of serazine (0.1–0.2 mL/kg). The hair around the knee joint were shaved and the surgical site was repeatedly cleaned with saline and hydrogen peroxide. After disinfection, anterior lateral incision of the knee joint was performed. The subcutaneous tissue was cut and the deep fascia and joint capsule were cut along the lateral edge of the patellar tendon. Then the patellar tendon was pushed to the medial side, followed by removal of the infraorbital fat pad and breaking of the transverse ligament of the knee. Finally, the knee joint was over-flexed to expose the lateral meniscus. The blood supply and the synovial membrane around the meniscus were cut with a No. 11 surgical blade to establish a model of full-thickness defect (1 mm × 4 mm) with no blood vessel in the inner 2/3 of the body and anterior horn of the lateral meniscus. In the AMF group, the autologous meniscus was cut into smallest pieces that can be manually processed (0.5 mm × 0.5 mm × 0.5 mm); these were then wrapped in the autologous myofascial and stuffed in the defect. Then the autologous myofascial membrane was continuously sutured with 3-0 polypropylene sutures and fixed on the meniscus. Lateral edge of the meniscus was sutured to the meniscotibial ligament at the distal portion and the meniscofemoral ligament at the proximal portion with the pullout technique, wherein each suture penetrated the meniscotibial and meniscofemoral ligaments and was knotted outside the joint capsule. The transverse knee ligament and joint capsule were closed with 3-0 polypropylene sutures. The incised patellar tendon and the skin wound were closed in layers with 3-0 nylon sutures. In the control group, the meniscus tissue was removed with a No. 11 surgical blade to establish a model of full-thickness defect (1 mm × 4 mm) with no blood vessel in the inner 2/3 of the body and anterior horn of the lateral meniscus, and then implanted in situ in the defect wrapped in an autologous myofascial sheath. Subsequently, each layer was repaired in the same manner. After surgery, all rabbits were returned to their cages and allowed to move freely without joint immobilization. Penicillin was injected 200,000 units/day on the 1st day after surgery. Rabbits who developed diarrhea were injected or fed with gentamicin sulfate 1–2 mL/time. If the rabbit died or the joint cavity infected, it should be supplemented accordingly to the relative group. The specimens were obtained along the original incision after the rabbits were euthanized at 2 weeks, 4 weeks, 8 weeks, and 12 weeks after operation.

### Gross observation

Macroscopic examination of the meniscus was performed as described by Ruiz-Ibán [28]. Briefly, the regenerative meniscus was classified into three grades, i.e., no healing, partial healing, and complete healing [28].

### Histological evaluation

All samples were fixed with 4% paraformaldehyde after gross observation. Subsequently, samples were embedded in paraffin and further cut into 6-μm thick sections. Hematoxylin and eosin (H&E) staining and immunohistochemistry staining for PCNA, COL1A1, and COL2 were performed to analyze the histology of regenerative meniscal tissue. A semiquantitative scoring system for the regenerative meniscus was applied as described by Narita et al.; the maximum score was 5 points [29]. All images were obtained using Phmias2008 ver 3.0 Demo (Phenix). The grey values of immunohistochemistry were analyzed using Motic images Advanced 3.2 (Motic).

### Statistical analysis

The continuous variables are presented as mean ± standard deviation (SD) and statistically analyzed using SPSS 23.0 (SPSS Inc., Chicago, IL, USA). The data of gross observation of the degree of healing degree and histological semi-quantitative scores were counted grade data. For gross observation and semi-quantitative scoring system, nonparametric test to rank transformation Mann–Whitney *U* was used to compare the differences between the control group and the AMF group at the same time-point, whereas nonparametric test to rank transformation Kruskal–Wallis *H* was used for comparisons among different time-points in the same group. For grey values, the homogeneity of variance of population means was tested first; then the comparison between the control group and the AMF group at the same time-point after surgery was performed using the independent sample *t* test or the *t* test for adjusting the degree of freedom when the variance was not equal. When the variance was equal, SNK-q was used to compare the differences among different time-points in the same group. When the variance was not equal, Dunnett’s T3 multiple comparison method or mixed model method was used. P values < 0.05 were considered indicative of statistical significance.

## Results

### Gross evaluation and scoring

**Fig. 2:**
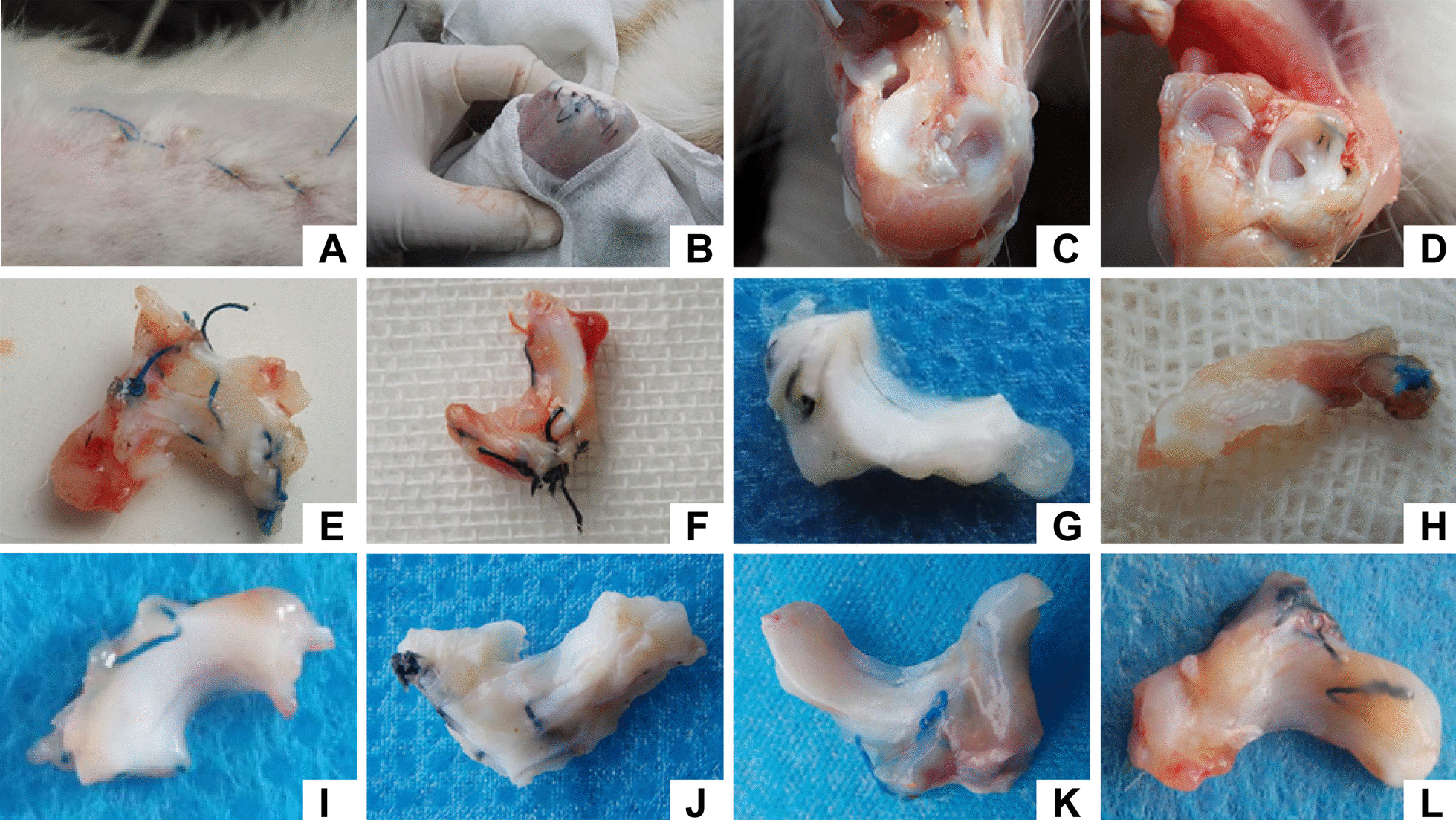
Macroscopic results of the repaired menisci. **A** Incision is not infected while obtaining specimen; **B** subcutaneous suture of the joint capsule shows no signs of infection; **C** normal rabbit knee meniscus; **D** 12-week knee joint specimen in the autogenous meniscal fragments (AMF) group; **E** no signs of healing at 2 weeks in the control group; **F** the defect is partially healed at 2 weeks in the autogenous meniscal fragments (AMF) group; **G** the defect is partially healed at 4 weeks in the control group; **H** the defect is partially healed at 4 weeks in the autogenous meniscal fragments (AMF) group; **I** the defect is partially healed at 8 weeks in the control group; **J** the defect is totally healed at 8 weeks in the autogenous meniscal fragments (AMF) group; **K** the defect is partially healed at 12 weeks in the control group; **L** the defect is totally healed at 12 weeks in the autogenous meniscal fragments (AMF) group

Six rabbits died while two left knees and one right knee developed infection after the surgery. The rest of rabbits showed good healing with no incidence of infection. The limb activity was slightly restricted, and all rabbits resumed normal activities and eating habits after 1 to 2 weeks. None of the rabbit knee joints included in the evaluation at 2, 4, 8, and 12 weeks after surgery showed infection at the surgical site (Fig. 2). There was no cartilage degeneration in the femoral and tibial articular surfaces; only some knee joints showed hyperplasia of the synovial tissues. Macroscopic observation showed that all transplanted meniscus tissues had survived, and there was no dissolution or necrosis in either of the groups (Table 1, Fig. 2).

**Table 1:** Gross observation of the menisci

Time (weeks)	Control (n = 8)			AMF (n = 8)		
	Total union	Partial union	Non-union	Total union	Partial union	Non-union
2	0	0	8	0	1	7
4	0	1	7	0	6	2
8	0	2	6	2	5	1
12	0	3	5	7	1	0

(1) In Control group: P = 0.264 > 0.05 at all-time points; Kruskal–Wallis H test

(2) In autogenous meniscal fragments (AMF) group: P = 0.000 < 0.05 at all-time points; Kruskal–Wallis H test; 2 weeks vs. 4 weeks, 8 weeks and 12 weeks, P = 0.015, 0.004, and 0.000; 4 weeks vs. 8 weeks and 12 weeks, P = 0.199 and 0.001; 8 weeks vs. 12 weeks, P = 0.009; Mann–Whitney U test

(3) Control vs. autogenous meniscal fragments (AMF) group: 2 weeks, P = 0.317 > 0.05; 4 weeks, P = 0.015 < 0.05; 8 weeks, P = 0.011 < 0.05; 12 weeks, P = 0.010 < 0.05; Mann–Whitney U test

Two weeks after surgery, fascia tissue was observed in both groups. The fascia tissue was reddish and showed potential blood supply. Some specimens showed signs of blood supply even at 4 weeks; however, the fascia was white in color and was tightly connected to the meniscus. Gross observation of meniscus specimens with non-union, partial union, and complete union in the two groups at 2, 4, 8, and 12 weeks after operation are shown in Table 1. At 4 weeks after operation, specimens in the AMF group showed signs of partial healing with observation of traces at the junction of the transplanted fragments. At 8 weeks after operation, some specimens showed disappearance of the traces of the junction of the transplanted fragments. At 12 weeks after operation, 7 specimens the traces of the junction of the transplanted fragments had completely disappeared and could not be identified. The regenerated tissues appeared like meniscal cartilage and were indistinguishable from the surrounding meniscal tissue.

As shown in Table 1, the degree of healing in the control group at each successive time-point was not statistically significant (*P* > 0.05). In contrast, the degree of healing in the AMF group showed a significant increase over time (*P* < 0.05); however, there was no significant difference in the degree of healing between 4 and 8 weeks after surgery (*P* > 0.05). There was no significant difference in gross grades between the AMF group and control group at 2 weeks after surgery (*P* > 0.05); however, the AMF group showed higher gross scores than the control group at 4, 8, and 12 weeks after surgery (*P* < 0.05).

### Histological evaluation and scoring

**Fig. 3:**
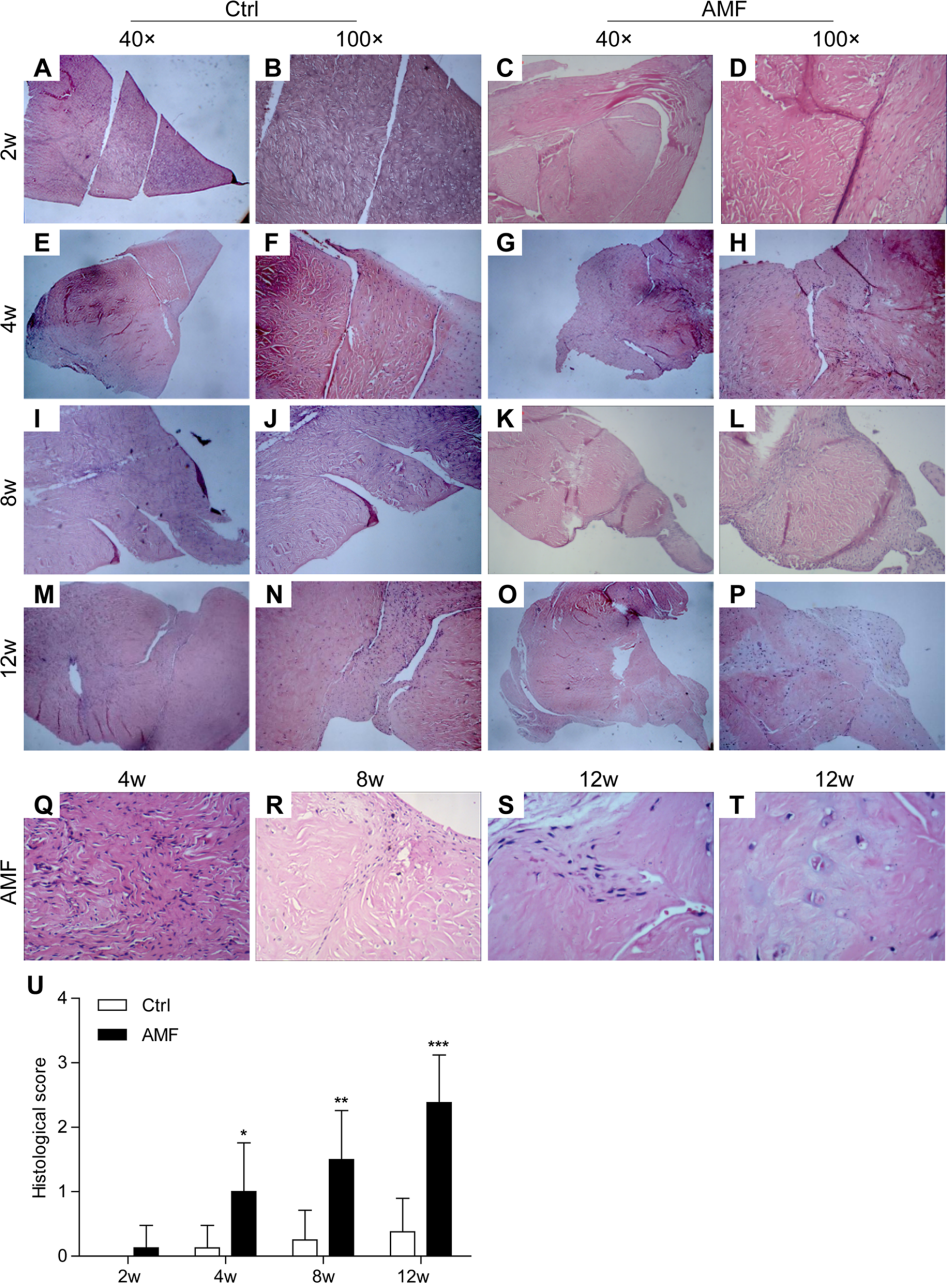
Representative HE staining and the histological score of the repaired menisci. **A**, **B** Control group at 2 weeks, score = 0; original magnification: **A** 4 × 10, **B** 10 × 10. **C**, **D** autogenous meniscal fragments (AMF) group at 2 weeks, score = 1; original magnification: **C** 4 × 10, **D** 10 × 10. **E**, **F** Control group at 4 weeks, score = 1; original magnification: **E** 4 × 10, **F** 10 × 10. **G**, **H** autogenous meniscal fragments (AMF) group at 4 weeks, score = 2; original magnification: **G** 4 × 10, **H** 10 × 10. **I**, **J** Control group at 8 weeks, score = 1; original magnification: **I** 4 × 10, **J** 10 × 10. **K**, **L** autogenous meniscal fragments (AMF) group at 8 weeks, score = 3; original magnification: **K** 4 × 10, **L** 10 × 10. **M**, **N** Control group at 12 weeks, score = 1; original magnification: **M** 4 × 10, **N** 10 × 10. **O**, **P** autogenous meniscal fragments (AMF) group at 12 weeks, score = 3; original magnification: **O** 4 × 10, **P** 10 × 10. **Q** Fusiform, polygonal immature fibrocartilage-like cells are seen at 4 weeks in the autogenous meniscal fragments (AMF) group; original magnification: 20 × 10. **R** Dense large round, elliptical cartilage-like cells are seen at 8 weeks in the autogenous meniscal fragments (AMF) group; original magnification: 20 × 10. **S** Mature fibrocartilage-like cells have healed the defect at 12 weeks in the autogenous meniscal fragments (AMF) group; original magnification: 20 × 10. **T** Chondrocyte lacuna can be found in the regenerative tissues at 12 weeks in the autogenous meniscal fragments (AMF) group; original magnification: 20 × 10. **U** Histological score of the repaired menisci. *P < 0.05, **P < 0.01, ***P < 0.001, compared with the control group

H&E staining and the semi-quantitative scores (Fig. 3) showed that the newly formed tissues gradually assumed characteristics akin to those of normal menisci. However, there was no significant difference in the semi-quantitative scores between the different time-points in the control group; in contrast, the scores increased over time in the AMF group (*P* < 0.05). Moreover, the newly formed tissues comprised of fusiform, polygonal immature fibrocartilage-like cells at 4 weeks, dense large, round, elliptical cartilage-like cells at 8 weeks, and mature fibrocartilage-like cells in the lacuna at 12 weeks. There was no significant difference between the AMF and control group with respect to the semi-quantitative scores at 2 weeks (*P* = 0.334). However, the semi-quantitative scores in the AMF group were significantly higher than those in the control group at 4, 8, and 12 weeks after surgery (*P* < 0.05). In the experimental group, the H&E results showed appearance of signs of repair on the surface of injured meniscus at 4 weeks after the operation, with proliferation of the same type of cells on the surface. The cells on the surface were spindle-shaped, with high cell density, whereas inner layer cells were fusiform, polygonal, naive fibrocartilage-like. At 8 weeks after operation, the repair range of the meniscus defect was gradually increased. The fissure site was filled with dense large circular or elliptical fibrocartilage-like cells. The cell density was significantly greater than that of the surrounding normal area. At 12 weeks after operation, the mature fibrocartilage-like cells were repaired, and chondrocyte lacunae were seen in the partially repaired area; in addition, the cell density and distribution were also akin to that of the surrounding normal area. Compared with the control group, the repaired area and the degree of repair in the experimental AMF group were significantly higher at 4, 8, and 12 weeks after surgery (P < 0.05).

### Cell proliferation in the regenerative menisci

**Fig. 4:**
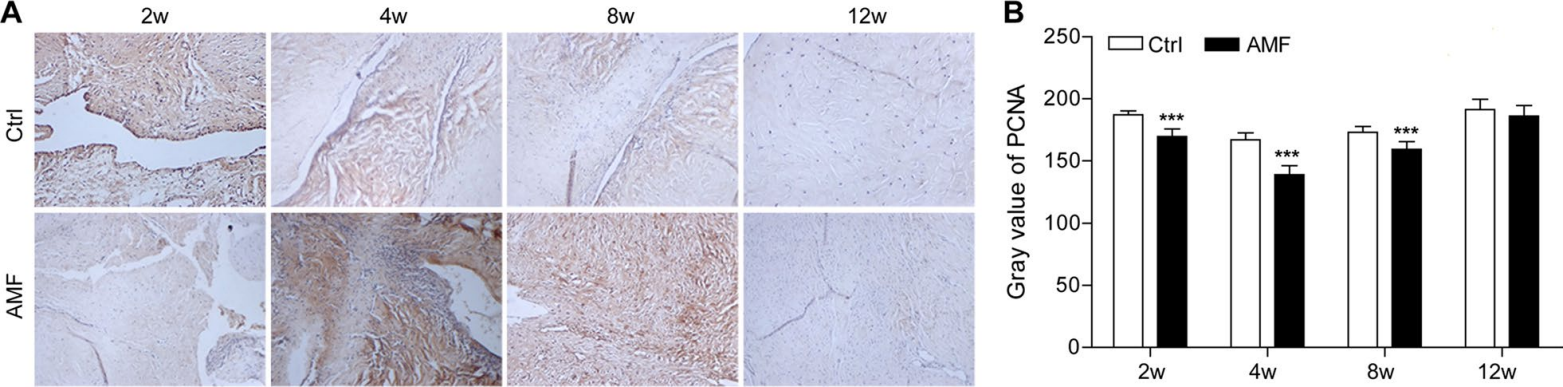
**A** Representative proliferating cell nuclear antigen (PCNA) immunohistochemical staining and **B** the proliferating cell nuclear antigen (PCNA) grey values of the repaired menisci in different groups at different time-points. Original magnification: 10 × 10. N = 8, mean ± standard deviation. ***P < 0.001, compared with the control group

To further evaluate the proliferation of the newly formed tissues after the implantation, we performed immunohistochemical assay for PCNA (Fig. 4) [30]. The magnitude of the mean gray value is inversely related to the PCNA protein expression level. The results of PCNA immunohistochemistry in the regeneration site showed that the average gray value of the AMF group was lower than that of the control group at 2, 4, and 8 weeks after surgery (*P* < 0.05). There was no significant difference in the mean gray value between the two groups at 12 weeks after operation (*P* > 0.05). The average gray value in the experimental group was the lowest at 4 weeks after surgery, and gradually increased at 8 weeks and 12 weeks after surgery.

### Extra cellular matrix (ECM) of regenerative menisci

**Fig. 5:**
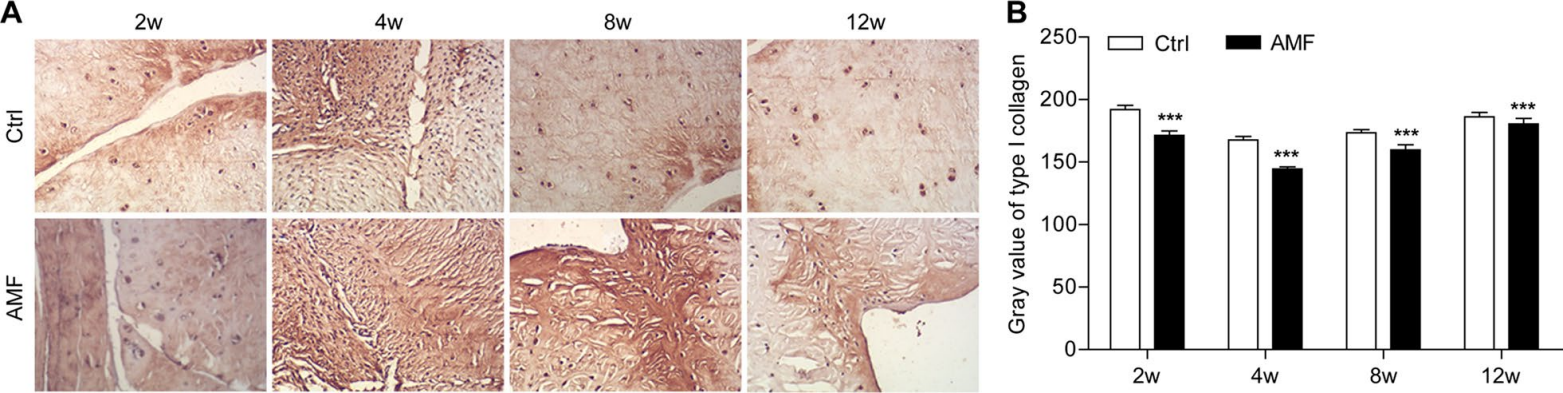
**A** Representative collagen type I (COL1A1) immunohistochemical staining and **B** collagen type I (COL1A1) grey values of the repaired menisci in different groups at different time-points. Original magnification: 10 × 10. N = 8, mean ± standard deviation. ***P < 0.001, compared with the control group

**Fig. 6:**
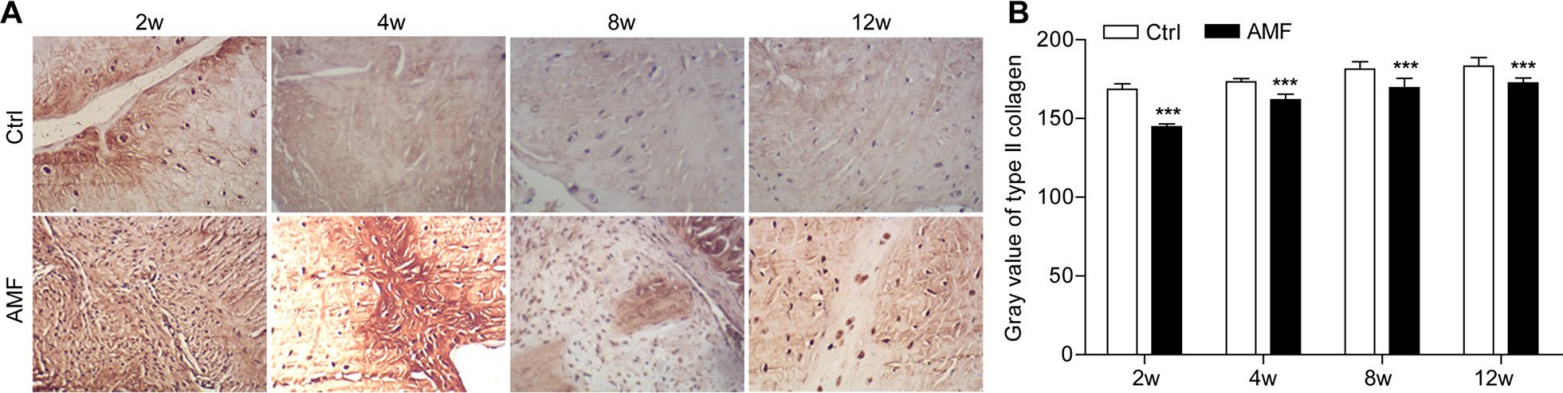
**A** Representative collagen type II (COL2A1) immunohistochemical staining and **B** collagen type II (COL2A1) grey values of the repaired menisci in different groups at different time-points. Original magnification: 10 × 10. N = 8, mean ± standard deviation. ***P < 0.001, compared with the control group

In the outer 2/3 region of the meniscus, COL1A1 (Fig. 5) accounts for 80% by dry weight. In the inner 1/3 region of the meniscus, COL2 (Fig. 6) accounts for 60% by dry weight and type I collagen accounts for 40% by dry weight [2] and are sensitive indicators of the proliferation and differentiation of meniscal fibrocartilage cells [23]. To assess the ECM of the regenerative menisci, we detected the above two types of collagen using immunohistochemistry. The average gray values of COL1A1 and COL2 in the AMF group were lower than those in the control group at each time-point (*P* < 0.05). At 2 weeks and 4 weeks after surgery, the expression level of COL1A1 increased in both the control group and the AMF group. However, the expression level of COL1A1 gradually decreased after 8 weeks. In addition, at 2, 4, 8, and 12 weeks after surgery, the immunohistochemical results showed a progressive decrease in the protein expression of COL2 in the AMF group and the control group at each successive time-point after operation. Whereas, at 8 and 12 weeks after surgery, there was no significant difference in the expression of COL2 in both the control group and the AMF group (*P* > 0.05).

## Discussion

Knee joint meniscal injury involving the avascular zone is commonly encountered in clinical settings; in such cases the damaged area cannot regenerate to form new meniscal tissue. The lack of self-repair ability of the damaged tissue further aggravates the structural damage, altering the biomechanics of the knee joint. The traditional treatment for injury of meniscal avascular zone is arthroscopic partial meniscectomy [31], which tends to lead to secondary osteoarthritis [32]. Therefore, it is advocated to maintain the anatomical integrity of the meniscus as far as possible rather than to remove the injured meniscus. In the present study, we found that AMF implantation significantly increased the repaired area and the degree of healing of meniscus at 4, 8, and 12 weeks after operation compared with the control group. In addition, the expression of PCNA (an important marker of cell proliferation state) was significantly upregulated in the AMF group. Similarly, the expressions of COL1A1 and COL2, which are the predominant components of ECM in the inner two thirds of the meniscus, were also higher than those in the control group. Besides, the expression of PCNA was not significantly different between the two groups at 12 weeks after operation. This implies that the proliferation level of the cells was low, which may have reached its limit and the unhealed tissue could not be repaired any more. This may provide a basis for determining the clinical time window of meniscus injury healing. Finally, these results suggest that the AMF method can promote the repair of rabbit meniscal injury in the avascular zone, and it has potential for clinical application.

Pathological studies have shown that the conditions for tissue damage repair include (1) cells involved in repair have intrinsic repair ability; (2) the defect area should have a scaffold structure; (3) cells growing into the defect area can continuously increase and (4) synthesize extracellular matrix [33]. The results of H&E staining showed that the repaired area and the degree of repair in the AMF group were significantly higher than that in the control group at each successive time-point after operation. This may be attributable to both the survival of the transplanted meniscus fragments and their acting as a scaffold in the defect area. Moreover, the autologous chondrocytes can proliferate, differentiate, and secrete the extracellular matrix. Compared with the control group, the autografted fragment tissue had greater contact area with the fissure site and the extracellular matrix secreted by the chondrocytes, allowing greater contact between the newly-proliferated cells and the fissure site. On the other hand, under stress and surgical stimulation, fibroblasts, chondroblasts, fibrocartilage cells, and a small amount of granulation tissue transformed by synovial-derived mesenchymal stem cells can be wrapped by myofascial tissue, thereby avoiding rinsing with synovial fluid to help promote damage repair.

Cisa et al. [34] used autologous synovial flap to fill a 6-mm-long full-thickness longitudinal injury in the medial meniscus with established angiogenesis. In this case, the implantation of the synovial flap served as a scaffold to enhance tissue regeneration. Moreover, the blood vessels at the synovial membrane grew into the injury site, providing nutrients, cell growth factors, and synovial-derived MSCs for tissue repair. Moreover, synovial MSCs derived from synovial membrane can transform into fibroblasts, chondrocytes, and fibrocartilage cells under stress and surgical stimulation, which plays a role in the repair of meniscus tissue [35]. Okuda et al. [15] reported that grinding the meniscus may stimulate the production of growth factors IL-1alpha, TGF-beta, PDGF, and PCNA [16], thus inducing repair of the medial meniscus in rabbits. However, this damages the normal meniscal structure, resulting in a decline in the overall function of the meniscus. Ruiz-Ibán et al. [28] also demonstrated the positive effects of fat-derived MSCs on healing the ruptures in the avascular zone of the medial meniscus of New Zealand white rabbits. Other studies have shown that stem cell transplantation can contribute to the healing of meniscal injury [31, 36]. However, use of stem cell transplantation for clinical treatment of meniscus damage in the avascular area is expensive. It has been reported that the degradable gelatin hydrogel complex platelet-rich plasma, basic fibroblast growth factor, connective tissue growth factor, and bio-protein glue can promote the healing of avascular areas of the meniscus [17]. The foregoing method for promoting meniscus repair can satisfy the conditions of injury repair by (1) activating the intrinsic repair potential of the damaged area or providing repair cells for the damage, (2) providing a scaffold structure for repair of tissue, and (3) promoting the proliferation of cells in the repair zone and the synthesis of extracellular matrix. In the future, surgeons may be able to take meniscus tissue from the edge of the injured meniscus site and process it into pieces and then transplant it. Therefore, fibrocartilage fragment grafting is a better choice.

Clinically, good results have been reported with the use of cartilage fragment grafting and I/III collagen membrane covering (ACI-C) to repair articular cartilage defects [37]. However, there is no clinical report pertaining to implantation of pieces of autologous meniscus for repair of meniscal injury in the avascular area; even basic researches are extremely rare. Even though there are differences in the structural characteristics of human and rabbit menisci, the distribution characteristics of the rabbit meniscus vessels make it more suitable to construct a model of healed meniscal injury in the avascular zone [38–40]. In this study, we implanted the autogenous meniscal fragments in the avascular zone (the inner 2/3 of the body and anterior horn of the lateral meniscus) in a rabbit model. Yasukazu et al. [25] transplanted meniscal fragments wrapped in autologous fascia into the defect site; they found that the fascia-wrapped meniscal fragments can significantly enhance the regeneration of fibrocartilage at the meniscal injury site. Therefore, in this experiment, we designed a 1 mm × 4 mm defect model in the avascular zone in the inner 2/3 of the body and anterior horn of the lateral meniscus of New Zealand white rabbits. The autologous meniscal tissue was processed into pieces (size: 0.5 mm × 0.5 mm × 0.5 mm) and transplanted into the defect site and sutured with myofascial wrap. The healing ability of the anterior horn of the meniscus that lacks blood supply was shown to be worse than that of the posterior horn [41]. Therefore, the design of the injury model of the meniscus was close to the anterior horn; in addition, the blood supply and structural characteristics of the rabbit meniscus are more suitable for establishing the injury model for avascular area. Our results show that autologous fragment graft can significantly promote the repair of damaged meniscus, and the wrapped fascial tissue may provide blood supply and nutrition for the healing of injured tissue in addition to fixation; this aspect needs to be further investigated in future.

In summary, the animal model established in this study offers the following advantages. First, the cost of the model are low, and the experimental operation is relatively simple. Second, in addition to increasing the degree of the meniscus tissue damage and stimulating the production of various cell growth factors, the transplanted meniscus fragment can also act as a scaffold facilitating the repair of the defect tissue; moreover, it increases the contact area between tissues, the migration ability of chondral cells, and the space for proliferation. AMF transplantation makes it easier for the tissue cells involved in the repair to absorb nutrients from the synovial fluid in the joint cavity without causing immune rejection. Thirdly, myofascial encapsulation of the fragments can stabilize the graft fragments, act as a fibrous scaffold, and prevent the graft tissue from being washed by joint fluid to promote the healing of the laceration. Certainly, the present study has some limitations that must be mentioned. First, the sample size of this study was small. Six rabbits died after surgery. The rabbits died due to various medical conditions, most commonly related to diarrhea caused by acute gastroenteritis and a perioperative mortality rate of 15.8%, although this rate is similar to that reported in the literature [42]. In future studies we will strengthen dietary hygiene management and reduce the incidence of acute gastroenteritis in New Zealand white rabbits. Second, in this study, we did not conduct allogeneic meniscus fragments graft. Potential clinical application in the future will typically require allografts. In this study, the acute phase injury of avascular area of meniscus was studied, but the chronic injury of meniscus was not studied. However, a large number of chronic meniscus injuries are encountered in clinical settings. In this study, the transplanted meniscus tissues were all fresh tissues, but in clinical practice, most patients with meniscus injury have degenerative tissues; it is uncertain whether the degenerative meniscus tissues still have the ability of proliferation and differentiation. Furthermore, we did not conduct biomechanical experiments after healing of specimens and did not assess whether the myofascial membrane itself has a role in angiogenesis. In the next step, we plan to repair the longitudinal fissure defect in the meniscal avascular zone area by using a mesh made of scaffold material to wrap biological protein glue in conjunction with autogenous meniscus fragments or autologous blood clot in conjunction with autologous meniscus fragments, and then conduct biomechanical tests after specimens healing. Our ultimate goal is to apply the technique of fragmentary transplantation to repair the longitudinal fissure of meniscus in humans. In addition, the related molecular biological mechanisms need to be further studied. In this study, we achieved great success in promoting healing of the avascular zone defect of the rabbit meniscus by grafting fragments; however, further studies are required to unravel the specific mechanisms. Further basic scientific research is required to elucidate the mechanisms that could enhance the healing potential and facilitate the development of new effective therapies for regeneration of damaged tissues.

## Conclusions

We established an animal model of repair of avascular zone defect of lateral meniscus in New Zealand white rabbits. As a basic practical research, the method of transplantation of fragments to repair the injury of adult rabbit meniscus in the avascular area provides a new strategy for clinical treatment of meniscal injury involving the avascular area. Further research may help pave the way for eventual clinical application of this technique. Compared with the method of one-piece meniscal implantation, the AMF method can better promote the repair of meniscal injury in the avascular zone, indicating that the AMF method may be used in clinical applications.

## Supplementary Information

**Additional file 1: Figure S1. **The details of the defect model.

## Data Availability

The datasets generated and analyzed during the present study are available from the corresponding author on reasonable request.

## Abbreviations

AMF: Autogenous meniscal fragments
ECM: Extra cellular matrix
MSCs: Mesenchymal stem cells
PCNA: Proliferating cell nuclear antigen
COL1A1: Collagen type I
COL2: Collagen type II
SD: Standard deviation
IL-1alpha: Interleukin-Ialpha
TGF-β: Transforming-growth-factor-beta
PDGF: Platelet-derived growth factor
