# Supplementary figures and images for: Regeneration of meniscal avascular zone using autogenous meniscal fragments in a rabbit model
Source: BMC Surg. 2022 May 28;22:209. doi: 10.1186/s12893-022-01663-3 (PMC9148493; doi:10.1186/s12893-022-01663-3)

Figure S1. The details of the defect model.


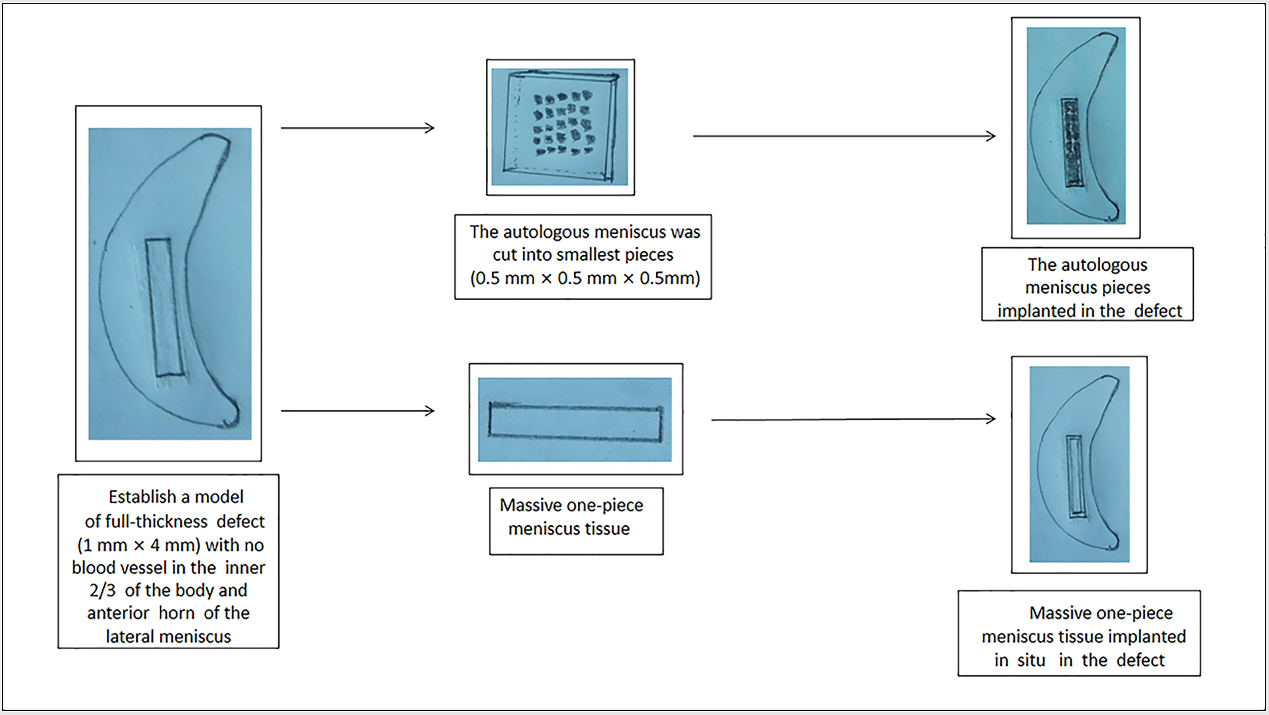

Supplement: Supplementary file 1 — Additional file 1: Figure S1. The details of the defect model. [file 12893_2022_1663_MOESM1_ESM.docx]
